# Accomplishing dietary and biochemical standards and improving hemodialysis efficiency with a non-compliant  patient; a case study

**DOI:** 10.12861/jrip.2014.22

**Published:** 2014-07-01

**Authors:** Chaudhary-Muhammaed Junaid-Nazar, Manzoor A. Lala, Saba Izhar, Percy Joesph

**Affiliations:** ^1^Department of Medicine, University of Buckingham, Ealing Hospital, NHS Trust, London, UK; ^2^Rural Health Development Program, Rural Area Development Council, Punjab, Pakistan; ^3^Department of Medicine, Allamaiqbal Memorial Teaching Hospital, Sialkot, Punjab, Pakistan; ^4^Department of Medicine, University of Buckingham, Ealing Hospital, NHS Trust, London, UK

**Keywords:** Hemodialysis, Renal Disease, Diet, Renal Association Standards, End Stage Renal Failure

## Abstract

**Background:** It was not until mid nineties when UK (RAS) and US (K/DOQI) first launched the nutritional and biochemical standards for haemodialysis in patients with ESRF. The present case is related to a patient who’s blood results diverged widely from the nutritional and biochemical standards set by the RAS. And how the multidisciplinary team with this patient aimed to achieve these standards.

**Case:** A 52-year old, staff nurse presented with end stage renal failure due to polycystic kidney disease with bilateral nephropathy, established on haemodialysis unusual inter-dialytic weight gains, often severe intradialytic cramps and hypotension to the point of being unresponsive. The patient’s high weight gain and high serum potassium and phosphate levels led to the patient being labelled non-compliant. Other contributing factors together with weight gains have to be explored.

**Conclusion:** Renal health care professionals have guidelines which they can work with their patients. Outside target results should be investigated to ensure that patient receives the right treatment. Treatment modality and prescription have to be individualized according to the patient’s needs. Like this case it is worth considering other factors like events in the patient’s life cycle, personal, social and economical factors and staff’s attitude may contribute to the perceived non-compliance.

**Implication for health policy/practice/research/medical education:**

During renal dialysis, intradialytic blood results can diverge widely from the recommended standards. Renal health care professionals have now guidelines including nutritional and biochemical targets readily available towards which they can work with their patients. When a patient’s blood results or fluid/nutritional intake are consistently outside the targets, the reasons for this should be investigated to ensure that the patient receives the right treatment.

## Introduction

In early nineties, the UK’s renal association’s standard published the first UK Renal Association Standards (RAS, nutritional and biochemical standards for hemodialysis patients). In 1995 another standard group, DOQI (Dialysis Outcome Quality Initiative – later K/DOQI), was launched in the US by the national kidney federation and first published in 1997 (1,2). Both K/DOQI and RAS cover similar aspects of kidney disease, renal failure treatment and have come up with similar standards, but have small differences between guidelines. K/DOQI for example sets the standard for serum phosphate levels in pre-dialysis patients lower than the RAS (K/DOQI 0.87–1.49 mmol/l versus RAS< 1.9 mmol/l). This may be in the best interest of the patient, but it is questionable if such levels are realistically achievable without compromising a patient’s quality of life and nutrition too much. Since K/DOQI standards are sponsored by pharmaceutical companies, there may also be a degree of self interest involved. Since the publication of RAS and K/DOQI other countries have developed their own standards, such as the Australian CARI Guidelines or the European Best Practice Guidelines. This essay however will discuss the case of a patient whose blood results diverged widely from the nutritional and biochemical standards set by the RAS (1-4). The factors that may have led to this divergence and how the multidisciplinary team together with the patient aimed to achieve these standards. Due to the restraints of the word count this essay can only focus on the standards from which the patient diverged (1-3).

### Albumin

Low albumin is a reverse prognostic marker of survival in dialysis and post transplant patients and is also often used as an indicator of malnutrition (4). The RAS recommends the regular measurement of serum albumin and suggests a level of over 30–35 g/l, depending on the lab assay used. Lower levels should prompt a clinical assessment of the patient for fluid overload, malnutrition, under-dialysis or acute events, such as infections.

### Phosphate

High phosphate levels are a common problem among hemodialysis patients and contribute to cardiovascular disease and osteoporosis (3,4). High levels cause serum calcium to bind to it, leading to lower serum calcium levels as well as tissue and vascular calcium-phosphate calcifications (3). Low calcium levels lead to increased parathyroid hormone (PTH) production, which increases calcium resorption from the bone, resulting in osteoporosis. RAS recommends phosphate levels of under 1.8 mmol/l, higher levels are associated with higher mortality rates. Much lower levels are difficult to achieve without compromising adequate protein intake. Due to the characteristics of phosphate, dialysis is of limited help controlling its levels. High distribution volume and protein binding capacities make phosphate removal difficult and lead to rapid rebound after dialysis. Dietary control alone can lead to malnutrition; phosphate is tied to protein intake (2).

### Potassium

Hyper and hypokalemia can have serious cardiac consequences for patients (1-4). Hypokalemia can lead to arrhythmias, but is rare in chronic dialysis patients. The RAS suggest pre-dialysis potassium levels of 3.5–6.5 mmol/l. The use of low potassium dialysate and drugs causing an increase of serum potassium, such as ACE inhibitors, should be avoided.

### Dialysis frequency and dose

RAS suggest that hemodialysis patients should dialyze thrice weekly, either achieving a Urea Reduction Rate (URR) of over 65% or a Kt/V of over 1.2. Consistent levels below these tar-gets are associated with increased mortality levels and a decrease of general well being. Higher dialysis doses can be achieved in various ways, for example by increasing dialysis frequency and time, but patient acceptance and service constraints can be a problem. Other options are daily short dialysis or nocturnal dialysis over longer hours. They are however resource consuming. Logistic restraints make delivery of these options in a dialysis unit difficult; therefore they are mostly carried out by independent, stable patients with good social support in a home setting.

## Case Presentation

A 52 years female patient presented with ESRF due to polycystic kidney disease and bilateral nephrectomy, established on hemodialysis via a brachiocephalic-arteriovenous fistula. She had a usual interdialytic weight gains between 3.0 kg and 5.0 kg. She often suffer with severe inter-dialytic cramps and episodes of hypotension. Sometime these episodes showed severeness to the point of being unresponsive. Dialysis access was difficult to cannulate. Her average midweek pre dialysis blood results were, albumin 40–42 g/l; phosphate 2.5–3.2 mmol/l; potassium 5.8–7.8 mmol/l. The patient was a staff nurse and her colleagues in the same unit assumed she should be familiar with dietary and fluid status concepts. Theses pre assumptions and the patients high weight gains with high serum potassium and phosphate led to the patient being labelled non-compliant. Regardless whether such labeling was appropriate, other contributing factors to the high blood results and weight gain had to be explored. Under the initial treatment prescription, the initial hemodialysis prescribed 3 hours thrice weekly, using a mid flux dialyzed, blood flow of 250 ml/min, dialyze flow of 500 ml/min, achieving an aver-age KT/V of 0.7, which indicating under-dialysis. Cannulation problems of the dialysis access and the need to use single needle dialysis on occasion contributed to this. Target weight of the patient was set at 72 kg. The patient did not receive a sufficient does of dialysis, which may have contributed to her high serum potassium and phosphate levels. Three hours were also enough to deal with the patient’s fluid overload, high filtration rates also led to inter-dialytic hypotension. Changes were made to the treatment prescription without ensure the correction of the calculated Kt/V. Blood sampling errors can lead to erroneous results (4). Changes to the treatment prescription often involves an increase of dialysis time or frequency which was true in this case. The patient’s individual circumstances and quality of life should be considered and the possible effects of under dialysis and benefits of adequate dialysis have to be discussed with the patient to enable them to decide whether the positive effects outweigh the increased time spent on the machine. In this case, there was a also history of previous abdominal surgery due to which she was not suitable for peritoneal dialysis and home hemodialysis was not favored as an option by the patient herself.

## Discussion

There are many factors in the treatment prescription which can be changed to increase dialysis adequacy (1): In this context, a dialyzer change from mid to high flux allowing more effective clearance of urea, creatinine and phosphate. Because high flux dialyzers are more expensive but this has to be justified with a sufficient and necessary increase in Kt/V. High flux dialyzers are regarded as having a higher risk of endotoxin contamination due to back filtration and therefore require better water quality. There is however evidence that the thinner membranes of low flux dialyzers allow endotoxin contamination as well. Therefore, water quality should always be of the highest standard (4). An increase of blood flow to over 300 ml/min, sufficient access flow of over 500 ml/min and correct needle size are necessary to achieve this (2). Access stenosis or needles placed to close together can lead to increased re-circulation, especially with higher flow rates. The patient presented had various access problems preventing a reliable delivery of higher blood flows. Longer treatment hours are effective in increasing dialysis efficiency, but not always possible or favored by patients. The patient presented worked full time, but after discussion agreed to longer hours especially because of the resulting reduction of hourly ultrafiltration rate and possible prevention of intra-dialytic hypotension. Treatment time was gradually increased to 4 hours and in this case could be managed without having to change other patient’s treatment time or staff shift patterns. Increase of dialyzate flow increases dialysis efficiency slightly, but only in conjunction with high flux dialyzers and blood flow rates over 350 ml/min (2,5,6). The increase is however minimal and because of the severity of under dialysis in this case was not considered a valuable option.

### Access

Good dialysis access is vital for the delivery of efficient dialysis. Factors such as stenosis can lead to inefficient access blood flow, recirculation and access thrombosis (2). The patient presented had a deep brachiocephalic fistula. Due to its depth it was difficult to cannulate and on several occasions the patient had to be dialyzed via single needle, which reduces dialysis efficiency (3,7). High venous pressures suggested stenosis and recirculation and did not allow reliable achievement of sufficient blood flow rates. Recirculation tests showed a recirculation rate of over 20%, leading to vascular assessment where a stenosis was diagnosed and surgically resolved. At the same time the fistula was lifted and depth decreased. Subsequently cannulation was mostly successful and blood flow rates of up to 350 ml/min could be achieved reliably. Solving the access problems also reduced cannulation anxiety and resulting treatment resentment in the patient.

### Fluid balance and target weight

The patient suffered regularly severe intradialytic cramps and hypotension, attributed to high interdialytic weight gains. Excess fluid overload in hemodialysis patients is associated with left ventricular dysfunction and hypertrophy and connected higher cardiovascular mortality (2). Frequent intradialytic hypotension is associated with increased mortality rates, though it is not clear if this is a cause or an indicator of underlying cardiac disease (4). Addressing the patient’s chronic fluid overload was therefore important. As a first measure a recommendation for daily fluid allowance was established, using 24 hour urine collection and advising the patient to limit fluid intake to 24 hour output + 300 ml per day. The patient received also education regarding fluid contents of food. Dialysis patients undergo a complete change in life-style and much of the recommendations they are given is contradictory to recommendations for healthy individuals. Therefore it is important not only to educate and give advice, but also to check if the information given is understood in the intended way. Assessment of target weight was also necessary. Patients’ weights fluctuate over time and a target weight set too low can lead to intradialytic hypotension and cramps (2,3). The patient’s target weight was evaluated at each session and adjusted frequently, increasing by 6.0 kgs over a two months period, bearing the risk of fluid overload in mind. As a result the patient suffered less cramps and hypotensive episodes, suggesting target weight was initially set too low.

As an additional measure dialysate sodium was adjusted to plasma sodium. Dialysate sodium lower than plasma sodium can lead to cramps and hypertension due to sodium removal. Higher dialysate than plasma sodium increases the patient’s plasma sodium over the course of the treatment, leading to increased thirst (1,2). The patient could also not tolerate more than 700 ml/hr ultrafiltration. This made occasionally an increase of treatment time to 4 ½ hours necessary. Despite the longer hours, taking the above measures made treatment more tolerable for the patient and relieved her again of some treatment anxiety. When patients present with chronic fluid overload, other factors leading to increased thirst, such as diabetes and uremia have to be considered, but were not a problem in this case.

### Dietary assessment

The patient was asked by the dietician to keep a food diary. She agreed to do this, but was not very enthusiastic about it and perceived it as an intrusion. Even with compliant patients the accuracy of food diaries may be doubtful. Many patients admit they forget making entries or do not document some things because they are embarrassed.

Social factors played a role in this patient’s food intake. Because she lived on her own and worked full time she did not have much time to prepare her own meals, relying on ready meals and takeaways, which are potentially high in phosphate. The patient received dietary advice and education regarding her high potassium and phosphate intake, but other factors were considered too (4):

- Supplements, such as vitamin tablets or low salt, can contain potassium
- ACE inhibitors, prescribed for blood pressure control, inhibit potassium secretion through the bowel
- Recirculation in the fistula can lead to decreased potassium and phosphate removal during dialysis
- Adequate phosphate binders have to be prescribed in the right dose and the patient needs to be educated regarding the correct time of intake
- Hemodialysis patients need adequate protein intake, which automatically leads to higher phosphate intake

### Other contributing factors

Every effort was made to increase the patient’s dialysis efficiency, optimize her diet and fluid balance and improve comfort on dialysis. As a result, Kt/V remained stable above 1.2, but serum potassium and phosphate levels continued to be high, 6.0–7.0 mmol/l and 2.5– 2.8 mmol/l respectively. The patient gained less weight between sessions, but was still often overloaded with gains of up to 4.0 kg. It was assumed that the patient still made dietary mistakes and that further education and advice might be necessary. However, when the patient was told about the blood results, she showed no surprise. In conversations with the nurses and dietician she admitted to being non-compliant with her diet and that she fully understood the long and short term risks of high potassium and phosphate levels and fluid overload. When further advice was offered she declined, stating she felt she had enough education and wanted to be left alone. It emerged that during her initial adaptation to dialysis the patient suffered from depression and feelings of isolation from friends and family. Additionally, in short succession her father died and her ex-husband, with whom she had two sons, committed suicide. Though she did not have a close relationship with both men, she said she was more affected by those two deaths than she thought. Counseling offered was declined by the patient. At some stage she complained about the attitude of some staff members regarding her weight gains and dietary intake, saying she felt patronized. She explained that she fully understood the consequences of her actions, but that at the moment she did not have the energy or will to stick to dietary or fluid intake restrictions. Following this, staff members were re-educated to achieve an attitude change and to ensure that patients receive the advice they need as well as understanding when life circumstances make it difficult to be compliant with diet, fluid intake or medication.

## Conclusion

Renal health care professionals have now guidelines including nutritional and biochemical targets readily available towards which they can work with their patients. When a patient’s blood results or fluid/nutritional intake are consistently outside the targets, the reasons for this have to be investigated. One important issue is to ensure that the patient receives the right treatment. Treatment modality as well as treatment prescription have to be individualized to the patient’s needs and dialysis access has to be well functioning. Patients should receive education and advice regarding their fluid and dietary intake and their understanding should be ensured. The influence of medication has to be considered as well and if necessary medication needs to be adjusted. Once all these measures are taken, blood results, weight gains and dietary intake may still not be as desired by renal professionals. In the present case it was worth considering if other factors such as events in the patient’s life cycle, social factors or staff attitude may contribute to the perceived non-compliance. It is important that patient’s are informed about the long term effects of dietary and fluid intake mistakes, but on occasion compliance with the restrictions imposed upon them may be difficult. Under those circumstances understanding, patience and tolerance from renal staff are necessary. It is also worth considering that non-compliance is defined by health professionals setting an ideal standard. Patient’s however may carry out a cost-benefit analysis, weighing the benefits of the advice given against the risks not following it, then making a decision whether to comply or not (6). Personal and social factors play a large role in this decision making. Health professionals have to bear in mind that, after having received and understood the available advice, it is the right of patients to make their own decisions regarding their health and longevity.

## Authors’ contributions

CMJN and SI prepared the primary draft. LMA and PJ further edited the paper.

## Conflict of interests

There are no competing interests.

## Ethical considerations

Ethical issues (including plagiarism, informed consent, misconduct, double publication and redundancy) have been completely observed by authors.

## Funding/Support

Self funded.
